# The Effects of *swnH1* Gene Function of Endophytic Fungus *Alternaria oxytropis* OW 7.8 on Its Swainsonine Biosynthesis

**DOI:** 10.3390/microorganisms12102081

**Published:** 2024-10-17

**Authors:** Dan Li, Xinlei Zhao, Ping Lu, Yu Min

**Affiliations:** 1 College of Life Science and Technology, Inner Mongolia Normal University, Hohhot 010022, China; m17695475658@163.com (D.L.); wujnlazr@outlook.com (X.Z.); minyu0137@163.com (Y.M.); 2 Key Laboratory of Biodiversity Conservation and Sustainable Utilization in Mongolian Plateau for College and University of Inner Mongolia Autonomous Region, Hohhot 010022, China; 3 College of Life Science, Inner Mongolia University, Hohhot 010021, China

**Keywords:** SW, gene knockout, *A. oxytropis* OW 7.8, *swnH1* gene

## Abstract

The *swnH1* gene in the endophytic fungus *Alternaria oxytropis* OW 7.8 isolated from *Oxytropis glabra* was identified, and the gene knockout mutant Δ*swnH1* was first constructed in this study. Compared with *A. oxytropis* OW 7.8, the Δ*swnH1* mutant exhibited altered colony and mycelium morphology, slower growth rate, and no swainsonine (SW) in mycelia, indicating that the function of the *swnH1* gene promoted SW biosynthesis. Five differential expressed genes (DEGs) closely associated with SW synthesis were identified by transcriptomic analysis of *A. oxytropis* OW 7.8 and Δ*swnH1*, with *sac*, *swnR*, *swnK*, *swnN,* and *swnH2* down-regulating. Six differential metabolites (DEMs) closely associated with SW synthesis were identified by metabolomic analysis, with P450, PKS-NRPS, saccharopine, lipopolysaccharide kinase, *L*-PA, α-aminoadipic, and *L*-stachydrine down-regulated, while *L*-proline was up-regulated. The SW biosynthetic pathways in *A. oxytropis* OW 7.8 were predicted and refined. The results lay the foundation for in-depth exploration of the molecular mechanisms and metabolic pathways of SW synthesis in fungi and provide reference for future control of SW in locoweeds, which would benefit the development of animal husbandry and the sustainable use of grassland ecosystems.

## 1. Introduction

Locoweeds refer to some plants containing swainsonine (SW) from *Oxytropis* DC. and *Astragalus* L., which are distributed worldwide in Asia, North America, and Australia [1,2,3,4,5]. Prolonged ingestion of locoweeds by livestock leads to locoism, manifesting neurological impairment, infertility, abortion, and death in severe instances [4,6,7,8,9,10]. The spread of locoweeds results in substantial economic losses for the grassland livestock industry [4,11,12,13]. The toxic compound responsible for locoism is swainsonine (SW), an indolizidine alkaloid with a chemical structure resembling mannose [5,14]. SW inhibits the function of lysosomal α-mannosidase I and Golgi mannosidase II, disrupting protein synthesis and processing, which leads to cellular vacuolation [4,5,6,15,16]. SW in various locoweeds is synthesized by endophytic fungi identified as *Alternaria oxytropis* [1,17,18,19,20,21,22,23,24]. However, the molecular mechanism and biosynthesis pathway of the SW synthesis in endophytic fungi in locoweed are still unclear.

The *saccharopine reductases* (*sac*) gene (KY052048) and *swnN* gene (OR596336) in *A. oxytropis* OW 7.8 isolated from *Oxytropis glabra* were previously knocked out by our research group. Compared to OW 7.8, reduced levels of SW and saccharopine were observed in the knockout mutant Δ*sac*, while the *L*-lysine level remained relatively unchanged [25]. The SW concentration is higher in the *sac* gene complementation strain Δ*sac/sac* than in OW 7.8 and M1, indicating that the *sac* gene promotes SW synthesis in fungi. Compared to OW 7.8, reduced SW level was observed in the knockout mutant Δ*swnN*, while *L*-glutamate, α-ketoglutaric acid, and *L*-proline were up-regulated, and phosphatidic acid (PA) and 2-aminoadipic acid were down-regulated. SW was detected in the gene function complementation strain Δ*swnN/swnN*, indicating that the function of the *swnN* gene promoted SW biosynthesis [26]. Comparative genomics analysis was conducted on SW-producing fungi such as *Arthroderma otae*, *Metarhizium robertsii*, *Trichophyton equinum*, *Ipomoea carnea* endophyte (ICE), *A. oxytropis*, and *Pseudogymnoascus* sp., identifying the presence of the SWN gene clusters closely related to SW synthesis in these fungi [27]. The SWN gene clusters consist of *swnA*, *swnH1*, *swnH2*, *swnK*, *swnN*, *swnR*, and *swnT* (Table 1). However, five SWN genes, including the *swnR*, *swnK*, *swnN*, *swnH1*, and *swnH2* genes, exist in the *A. oxytropis* genome, while the *swnA* gene and *swnT* gene are absent [27,28]. The *swnH1* gene encoded 2-oxoglutarate/Fe(II)-dependent oxygenases (2OG oxygenases), of which one had non-heme-iron-containing enzymes that catalyze the oxidation of an organic substrate using ferric iron as the active site (R + 2OG + O_2_ → R–OH + succinate + CO_2_) [29,30,31,32,33,34]. SW was undetectable in *M. robertsii* when the *swnH1* gene was knocked out, while (1S/R,8aS) 1-hydroxyindoline and (1S,2S/R,8aS) 1,2-dihydroxyindoline were detected. It is postulated that the production of SW from (1R,2S,8aS)-1,2-dihydroxyindolizine or (1S,2S,8aS)-1,2-dihydroxyindolizine is catalyzed by the SwnH1 enzyme in *M. robertsii* [35].

**Figure 1:**
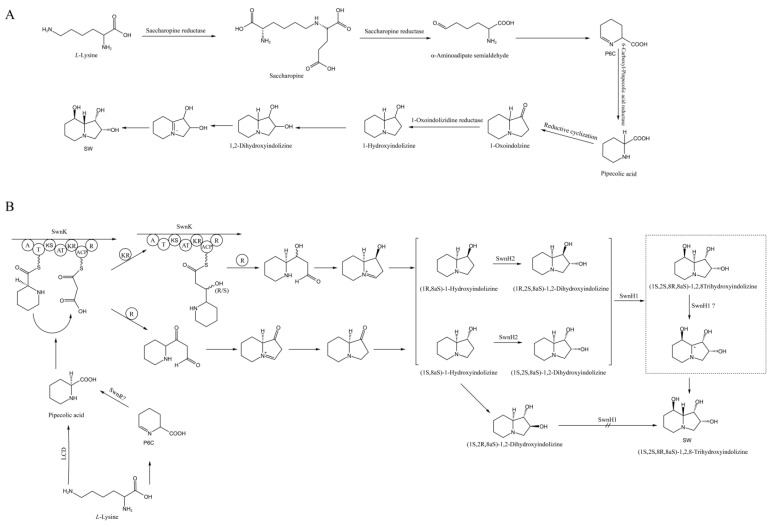
SW biosynthetic pathways in two fungi. (**A**) *R. leguminicola* and (**B**) *M. robertsii* [27,38].

Research on the SW biosynthesis pathway in fungi began with *Rhizoctonia leguminicola*, followed by in *M. robertsii* and *A. oxytropis* [27,35,36,37,38,39,40]. The predicted SW biosynthesis pathway in *R. leguminicola* is as follows: *L*-lys → saccharopine → α-aminoadipic semialdehyde → P6C → *L*-PA → 1-oxoindolizidine → 1-hydroxyindolizine → 1,2-dihydroxyindolizine → SW (Figure 1A) [37]. The predicted SW biosynthesis pathway from *L*-lysine to *L*-PA has two branches in *M. robertsii*: *L*-lys → *L*-PA and *L*-lys → P6C → *L*-PA. From *L*-PA to SW, there are also two branches: *L*-PA → 1-oxoindolizidine → 1-hydroxyindolizine → 1,2-dihydroxyindolizine → SW and *L*-PA → 1-hydroxyindolizine → 1,2-dihydroxyindolizine → SW (Figure 1B) [27]. The predicted SW biosynthesis pathway from *L*-lys to *L*-PA has two branches in *A. oxytropis*: *L*-lys ↔ saccharopine → α-aminoadipic semialdehyde → P6C → *L*-PA and *L*-lys → 6-amino-2-oxohexanoate → P2C → *L*-PA. The process from *L*-PA to SW is predicted to be *L*-PA → 1-hydroxyindolizine → SW [38].

In this study, the *swnH1* gene was firstly cloned and knocked out in *A. oxytropis* OW 7.8. The SW levels in the mycelia of the OW 7.8 and *swnH1* gene knockout strain Δ*swnH1* were determined. Additionally, the analyses of transcriptome and metabolome were conducted on *A. oxytropis* OW 7.8 and Δ*swnH1* to predict the SW synthesis pathway. The results lay the foundation for in-depth exploration of the molecular mechanisms and metabolic pathways of SW synthesis in fungi and provide reference for future control of SW in locoweeds, which will benefit the development of animal husbandry and the sustainable use of grassland ecosystems.

## 2. Materials and Methods

### 2.1. Strain

*A. oxytropis* OW 7.8 was isolated by our research group from *Oxytropis glabra* collected in Wushen banner, Ordos city, Inner Mongolia, China (108°52′ E, 38°36′ N, elevation 1291 m) [41]. The mycelia were cultured on PDA media (fresh peeled potato 200 g, glucose (KeMao, Tianjin, China) 20 g, agarose (LabLead, Beijing, China) 15 g, diluted to 1000 mL) at 25 °C.

### 2.2. Identification and cDNA Cloning of swnH1 Gene from A. oxytropis OW 7.8

The genomic DNA of *A. oxytropis* OW 7.8 was extracted (Plant Genomic DNA Kit, Tiangen, Beijing, China). Total RNA of *A. oxytropis* OW 7.8 was extracted (the OminiPlant RNA Kit, CWBIO, Shanghai, China) and reverse-transcribed to synthesize cDNA. The quality of the DNA and cDNA were verified by 1% agarose (LabLead) gel electrophoresis. Primers H1F (5′-ATGTCTCTATTTCCTCCGCCCA-3′) and H1R (5′-GCAAGGAACTGG TTAGGAATGGT-3′) were designed based on the genomic data of *A. oxytropis* OW 7.8 to amplify the *swnH1* gene (ON416998). The PCR program was as follows: 94 °C for 3 min; 94 °C for 30 s, 50 °C for 30 s, 72 °C for 1 min and 10 s, repeated for 30 cycles, and a final extension at 72 °C for 10 min (high-fidelity LA Taq enzyme (Takara, Beijing, China)). The PCR products were detected using 1% agarose gel electrophoresis, purified (SanPrep Column PCR Product Purification Kit, Sangon Biotech, Shanghai, China) and then sequenced (Sangon Biotech).

### 2.3. The Construction of the Phylogenetic Tree for the swnH1 Protein

The intergenic regions of the SwnH1 proteins for 15 SW-producing fungi were obtained from NCBI for *Alternaria oxytropis* Raft River (AQV04228), *Pyrenophora seminiperda* (RMZ73567), *Slafractonia leguminicola* (AQV04234), *Alternaria oxytropis* OW 7.8 (ON416998), *Metarhizium brunneum* (XP_014543162), *Metarhizium guizhouense* (KID83599), *Metarhizium majus* (KID96322), *Metarhizium robertsii* (XP_007824807), *Chaetothyriaceae* sp. (AQV04219), *Trichophyton benhamiae* (XP_003014119), *Trichophyton rubrum* (XP_003238865), *Trichophyton equinum* (EGE01987), *Trichophyton interdigitale* (EZF30351), and *Neonectria ditissima* (KPM44118.1) as the outgroup. The amino acid sequences were aligned by using the ClustalW algorithm, and the phylogenetic tree was constructed by using the neighbor-joining method in the MEGA11 software.

### 2.4. Construction of the swnH1 Gene Knockout Vector

**Figure 2:**
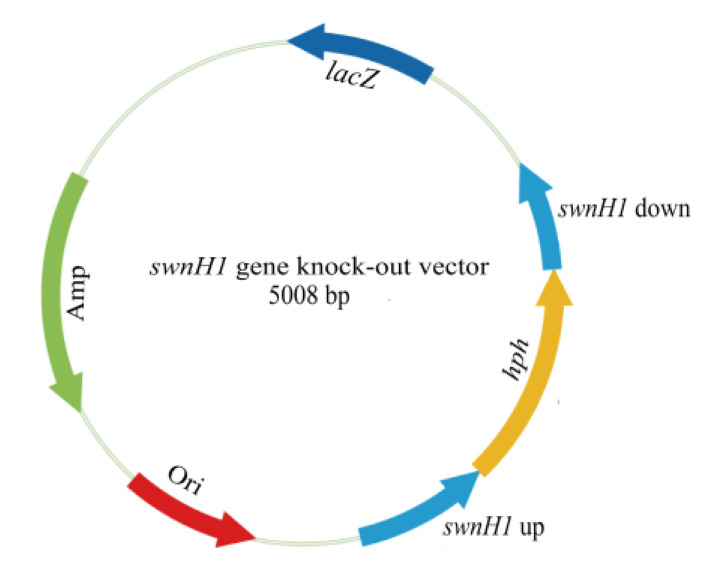
Diagram of the *swnH1* gene knockout vector structure. Amp: Ampicillin; Ori: Origin of replication; *swnH1* up: the upstream homologous sequences of *swnH1*; *swnH1* down: the downstream homologous sequences of *swnH1*; *hpt*: hygromycin phosphotransferase gene; *lacZ*: *lacZ* gene.

The upstream and downstream homologous sequences of the *swnH1* were amplified using the genomic DNA of *A. oxytropis* OW 7.8 as a template. The hygromycin phosphotransferase gene (*hpt*) was amplified using the pCB1003 plasmid (MiaoLingBio, Wuhan, China) as a template. The upstream and downstream homologous sequences of the *swnH1* were linked to the *hpt* gene on both sides using overlapping PCR to construct the *swnH1* gene knockout cassette, and this cassette was then ligated into the pMD^TM^-19T vector (Takara) using TA cloning to make up the *swnH1* gene knockout vector (Figure 2).

### 2.5. Sensitivity Test of A. oxytropis OW 7.8 to Hygromycin B

The mycelia of *A. oxytropis* OW 7.8 were inoculated on PDA media containing 0 μg/mL, 0.8 μg/mL, 0.9 μg/mL, 1 μg/mL, 2 μg/mL, and 3 μg/mL of Hygromycin B (Hyg B, Lab-lead, Beijing, China), respectively. The cultures were incubated in the dark at 25 °C for 30 days. The growth of colonies was observed, and an appropriate concentration of Hyg B was chosen to add to the media for *swnH1* gene knockout transformant screening.

### 2.6. Preparation and Transformation of A. oxytropis OW 7.8 Protoplasts

Preparation and transformation of protoplasts was performed as described by Hu et al. [42]. The mycelia of *A. oxytropis* OW 7.8 grown on PDA media were transferred into each 150 mL flask of PDB liquid culture media and incubated at 25 °C and 180 rpm for 5 days. The resulting mycelia were filtered through sterile miracloth. The collected hyphae were added with different concentrations of enzymatic hydrolysate (3% Driselase (Sigma, Shanghai, China), 1% Lysing enzyme (Sigma), 0.04% Chitinase (Sigma)) prepared by 1.2 mol/L MgSO_4_ (pH5.8, KeMao), and enzymatic hydrolysis at 30 °C, 80 rpm for 4 h. The enzymatically digested mixtures were filtered through a layer of sterile miracloth into a sterile 50 mL centrifuge tube, and the protoplasts were washed extensively with 1.2 mol/L MgSO_4_ and centrifuged at 4000 rpm for 5 min at room temperature. After discarding the supernatant, 5 mL of STC Buffer (1 mol/L Sorbitol (Sigma), 100 mmol/L Tris–HCl (OXOID), 100 mmol/L CaCl_2_ (KeMao)) was added and the protoplasts were gently resuspended. The mixture was centrifuged at 4000 rpm for 5 min. After discarding the supernatant, 1 mL of STC Buffer was added. Finally, protoplasts were adjusted to 2–5 × 10^6^/mL for subsequent experiments.

Approximately 5–10 μg of the linearized *swnH1* knockout vector was added to 15 mL centrifuge tube containing 2–5 × 10^6^/mL protoplasts, and incubated in an ice bath for 30 min without shaking. Then, 1 mL of SPTC (40% PEG 8000 (Sigma) dissolved in STC Buffer) were added to the tube (mixed thoroughly by inversion) and incubated at room temperature for 30 min without shaking. The protoplasts were added to 10 mL of Bottom Agar (0.3% yeast extract (OXOID), 0.3% acid hydrolyzed casein (Coollaber), 20% sucrose (KeMao), 1% Agar (BBI, Shanghai, China)) containing 50 μg/mL ampicillin (Lab-lead). After incubation at 25 °C for 12 h, the Top Agar (0.3% Yeast Extract, 0.3% acid hydrolyzed casein, 20% sucrose, 1.5% agar) containing 50 μg/mL ampicillin and 2 μg/mL Hygromycin B was added. Each single colony transformant grown on the plate was transferred to PDA media containing 2 μg/mL Hygromycin B after 7–10 d.

### 2.7. Screening and Identification of Gene Knockout Transformants

**Figure 3:**
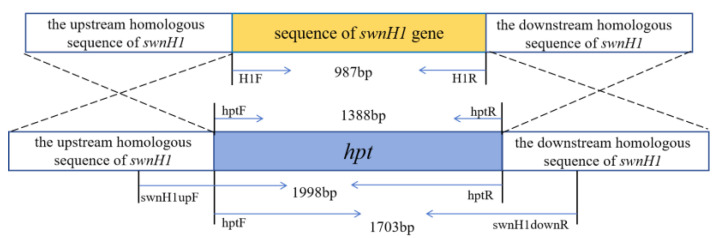
Identification figure for *swnH1* gene knockout transformants.

The *swnH1* gene knockout transformants were cultured on TB_3_ (0.3% yeast extract, 0.3% acid hydrolyzed casein, 20% sucrose) regeneration media (bottom layer: 50 μg/mL Amp and 1 μg/mL Hyg B; top layer: 50 μg/mL Amp and 2 μg/mL Hyg B) at 25 °C to cultivate. A single colony was transferred to PDA media (2 μg/mL Hyg B) for culture. Transformants resistant to Hyg B were screened and subjected to PCR analysis. The sequence upstream homologous to *swnH1* and *hpt* was amplified with primers swnH1upF/hptR (5′-GACCTATTCGGACCTACAAG-3′; 5′-GAACCCGCGGTCGGCATCTACTCTAT-3′). The sequence of *hpt* downstream homologous to *swnH1* was amplified with primers hptF/swnH1downR (5′-GCTGGAGCTAGTGGAGGTC-3′; 5′-GGTGCTGGCTACAACAC TATC-3′). The sequence of *hpt* was amplified with primers hptF/hptR (5′-GGCTTGGCTGGAGCT AGTGGAGGTC-3′; 5′-GAACCCGCGGTCGGCATCTACTCTAT-3′). The sequence of *swnH1* was amplified with primers H1F/H1R (Figure 3). All PCR products were sequenced for verification (Sangon Biotech).

### 2.8. Extraction and Detection of SW in Mycelia of A. oxytropis OW 7.8 and ΔswnH1 

Mycelia of *A. oxytropis* OW 7.8 and Δ*swnH1* cultured for 20 days were used for SW extraction by acetic acid/chloroform solution, purified by cation exchange resin, and SW solution was eluted by 1 mol/L ammonia solution. The SW levels in the mycelia were determined by HPLC-MS (G6430A, Agilent, Santa Clara, CA, USA), with three replicates for each sample [43]. All statistical analysis was conducted with GraphPad Prism 9.5.0 software with one-way ANOVA for each group of samples. The HPLC conditions were as follows: mobile phase, 5% methanol and 20 mmol/L ammonium acetate; flow rate, 0.4 mL/min; column temperature, 30 °C. MS conditions were as follows: positive ion, 156; negative ion, 70; IonSpray voltage (IS), 30V.

### 2.9. Colonies and Mycelia Morphology

The mycelia of *A. oxytropis* OW 7.8 were inoculated on PDA media containing 0 μg/mL of Hygromycin B (Hyg B), and the mycelia of Δ*swnH1* were inoculated on PDA media containing 5 μg/mL of Hygromycin B (Hyg B). The cultures were incubated in the dark at 25 °C for 20 days. 

Mycelia of *A. oxytropis* OW 7.8 and Δ*swnH1* strains cultured for 20 days were rinsed gently with PBS and quickly placed in the electron microscope fixative solution for 2 h. Fixed mycelia samples were rinsed with 0.1 mol/L phosphate buffer (pH 7.4) for 15 min, repeated 3 times, and fixed with 1% osmium acid in dark for 1 h. Samples were soaked in alcohol at different concentrations (30%, 50%, 70%, 80%, 90%, 95%, 100%) for 15 min and in isoamyl acetate for 15 min. The samples were placed in a critical point desiccator for drying. The dried samples were pasted on the double-sided tape of conductive carbon film and then the double-sided tape were placed on the sample stage of the ion sputtering apparatus for about 30 s. The mycelia morphology of *A. oxytropis* OW 7.8 and Δ*swnH1* was examined under a scanning electron microscope (SU81003.0kV × 30, Wuhan servicebio technology, Wuhan, China).

### 2.10. Transcriptomic Analysis of A. oxytropis OW 7.8 and ΔswnH1

Mycelia of *A. oxytropis* OW 7.8 and Δ*swnH1* strains (fresh weight 0.1–0.3 g) cultured for 20 days were rapidly frozen with liquid nitrogen in cryovials (1–3 min) and then delivered on dry ice with three replicates for each sample. Transcriptome sequencing was performed on the Illumina NovaSeq 6000 platform (Novogene, Beijing, China). The data were analyzed using DESeq2 [44] and clusterProfiler [43] package in R. The differentially expressed genes (DEGs |log_2_(Fold Change)| ≥ 1 and *p*-value ≤ 0.05) [42,44] were subjected to functional enrichment analysis with GO and KEGG [44,45,46].

### 2.11. Metabolomics Analysis of A. oxytropis OW 7.8 and ΔswnH1

Mycelia of *A. oxytropis* OW 7.8 and Δ*swnH1* (fresh weight 0.1–0.3 g) cultured for 20 days were rapidly frozen with liquid nitrogen in cryovials (1–3 min) and then delivered on dry ice with five replicates for each sample. Metabolome was performed using LC-MS (Novogene). The data were analyzed using Compound Discoverer package in R [45]. The differential expressed metabolites (DEMs VIP > 1, |log_2_(Fold Change)| ≥ 1 and *p*-value < 0.05) [46,47] were subjected to functional enrichment analysis with KEGG [47,48,49].

## 3. Results

### 3.1. Cloning and Bioinformatics Analysis of the swnH1 Gene in A. oxytropis OW 7.8

**Figure 4:**
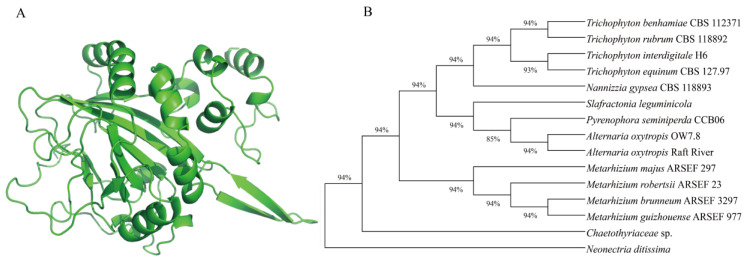
Bioinformatics analysis of the *swnH1* gene. (**A**) Predicted SwnH1 protein structure; (**B**) the phylogenetic tree of the SwnH1 protein.

The *swnH1* gene (GenBank: ON416998) in *A. oxytropis* OW 7.8 was cloned, with a length of 987 base pairs (bp, ATGTAG) and a 66 bp intron (631 bp697 bp). The length of *swnH1* cDNA is 921 bp (ATGTGA). The *swnH1* gene is predicted to encode a protein with 306 amino acids. The molecular formula of this protein is C_1624_H_2568_N_444_O_476_S_14_, with a molecular weight of 33.64 kDa and a pI of 5.94. The protein is hydrophilic (ExPASy–ProtScale), with no transmembrane domains (TMHMM Serverv 2.0) and no signal peptides (SignalP-6.0 Server). The advanced structure of the SwnH1 protein is predicted using the AlphaFold3 model (Figure 4A) [50]. It contains 33.99% α-helix (Hh), 44.06% random coil (Cc), and 18.95% extended chain (Ee).

A phylogenetic tree of SwnH1 proteins from 15 SW-producing fungi was constructed using the neighbor-joining method (Figure 4B) with 2 branches: One branch was divided into upper and lower branches. The upper branch consisted of four species of the genus *Trichophyton* and one species of the genus *Nannizzia.* In the lower branch, two species of *A. oxytropis* clustered first, then they clustered with *Pyrenophora semeniperda* and finally clustered with *Slafractonia leguminicola*. Another branch consisted of 4 species of *Metarhizium* clustered and one species of the genus *Chaetothyriaceae*. The amino acid sequence alignment of SwnH1 proteins shows that the highest sequence identity was 100% between *A. oxytropis* OW 7.8 and *A. oxytropis* Raft River. The sequence identities with *Pyrenophora seminiperda, S. leguminicola, Nannizzia gypsea,* and *Chaetothyriaceae* sp were 90.25%, 77.95%, 80.72%, and 78.03%, respectively. The sequence identities with the four *Metarhizium* species are approximately 77%, and the four *Trichophyton* species are approximately 80%.

### 3.2. Sensitivity Screening of A. oxytropis OW 7.8 to Hyg B

**Figure 5:**
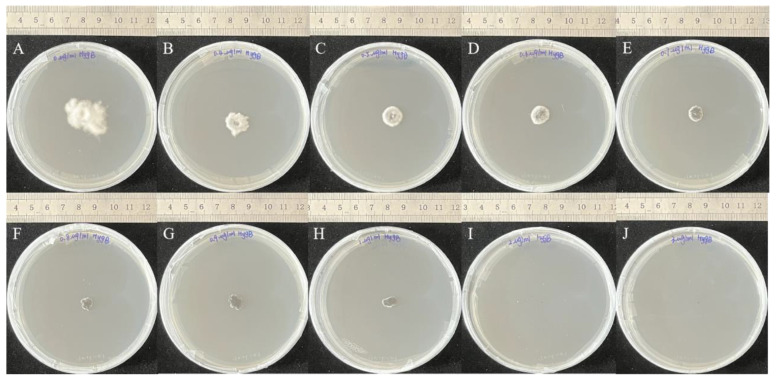
Colonies of *A. oxytropis* OW 7.8 on PDA media containing different concentrations of Hyg B after 30 days of incubation. (**A**) 0 μg/mL. (**B**) 0.4 μg/mL. (**C**) 0.5 μg/mL. (**D**) 0.6 μg/mL. (**E**) 0.7 μg/mL. (**F**) 0.8 μg/mL. (**G**) 0.9 μg/mL. (**H**) 1.0 μg/mL. (**I**) 2.0 μg/mL. (**J**) 3.0 μg/mL.

After incubation in darkness at 25 °C for 30 days, the growth of *A. oxytropis* OW 7.8 colonies on PDA media containing different concentrations of Hygromycin B (Hyg B) indicated that the fungus is sensitive to ≥2 μg/mL of Hyg B (Figure 5).

### 3.3. Identification for Transformants of ΔswnH1 Colonies

**Figure 6:**
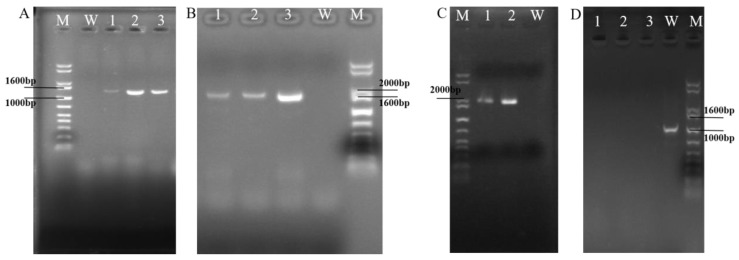
Electrophoresis analysis of PCR products of transformant DNA. Marker: 1 kb plus DNA ladder. (**A**) Lanes 1, 2, 3 show bands of the *hpt* gene, with the expected product being 1388 bp; W: negative control. (**B**) Lanes 1, 2, 3 show bands of the *hpt* gene + downstream homologous sequence of the *swnH1*, with the expected product being 1703 bp; W: negative control. (**C**) Lanes 1, 2 show bands of the upstream homologous sequence of *swnH1* + *hpt* gene, with the expected product being 1998 bp; W: negative control. (**D**) Lanes 1, 2, 3 show unamplified internal sequence of *swnH1*; W: positive control, with the expected product being 987 bp.

The *swnH1* gene knockout transformants gradually appeared on PDA media containing 2 μg/mL Hyg B one week after transformation. PCR results showed that bands of the *hygromycin phosphotransferase* gene, (*hpt*) gene, and *hpt* gene upstream homologous to the *swnH1* and the *hpt* gene downstream homologous to the *swnH1* were amplified in *swnH1* gene knockout transformants (Figure 6). The sequencing of the PCR products confirmed their accuracy, resulting in the identification of the Δ*swnH1*.

### 3.4. Morphology of Colonies and Mycelia

**Figure 7:**
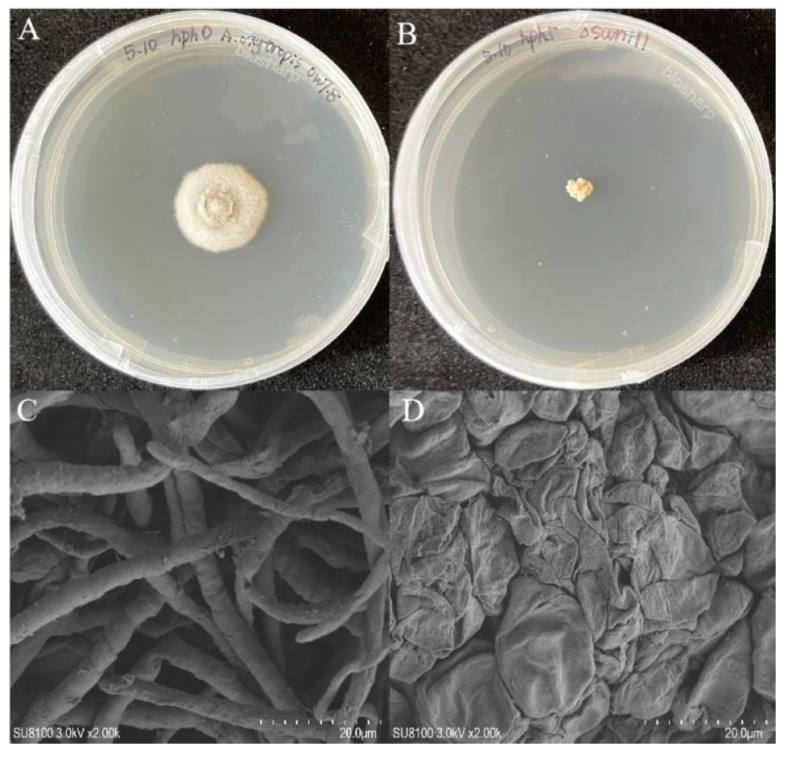
Morphology of colonies and mycelia from *A. oxytropis* OW 7.8 and Δ*swnH1.* (**A**) *A. oxytropis* OW 7.8 colonies. (**B**) Δ*swnH1* colonies. (**C**) *A. oxytropis* OW 7.8 mycelia magnified 3000×. (**D**) Δ*swnH1* mycelia magnified 3000×.

The in vitro cultured endophytic fungi displayed as round and raised, with uniform margin and radial growth colonies which grew slowly. Later, a black/brown pigment was secreted [24]. In contrast, the colonies of Δ*swnH1* appeared milky white or light yellow, with irregular shape and slow growth. The colonies were loose and granular, with no pigment accumulation, and the mycelia became enlarged with irregular morphology. Significant differences were observed in the mycelial morphology and structure between *A. oxytropis* OW 7.8 and Δ*swnH1* with scanning electron microscopy. Mycelia of *A. oxytropis* OW 7.8 exhibited typical single, tubular, smooth-surfaced and unbranched fungal hyphae. In contrast, Δ*swnH1* hyphae exhibited wrinkling and concavity (Figure 7).

### 3.5. SW Levels in the Mycelia of A. oxytropis OW 7.8 and ΔswnH1

**Figure 8:**
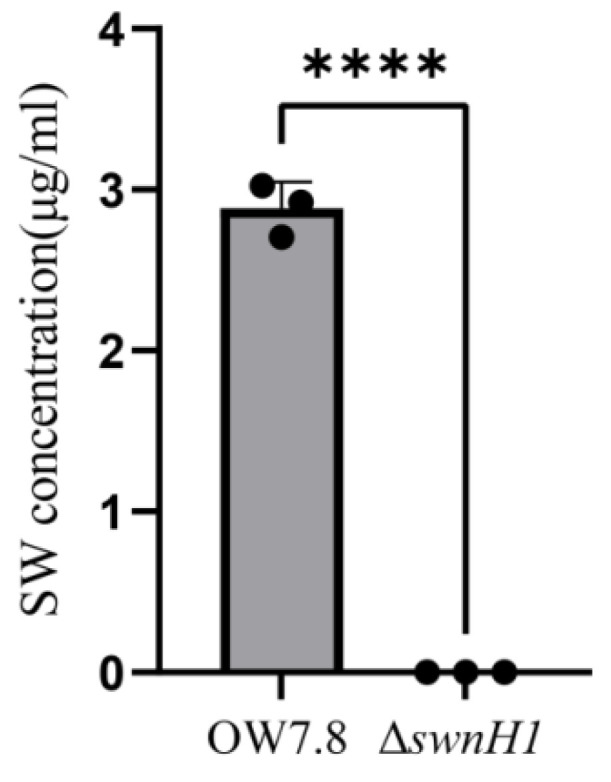
SW levels in mycelia from *A. oxytropis* OW 7.8 and Δ*swnH1*. Error bars represent the standard error of the mean (*n* = 3), with (****) *p* < 0.0001.

SW was not detected in the mycelia of Δ*swnH1*, whereas the SW level in the mycelia of *A. oxytropis* OW 7.8 was 2.884 ± 0.0949 μg/mL after 20 days of cultivation (Figure 8), indicating that the gene function of *swnH1* promotes SW synthesis. The regression equation for the SW standard curve was Y = 18.3759X + 2.4084 (R^2^ = 0.9927).

### 3.6. Transcriptome Analysis of A. oxytropis OW 7.8 and ΔswnH1

**Figure 9:**
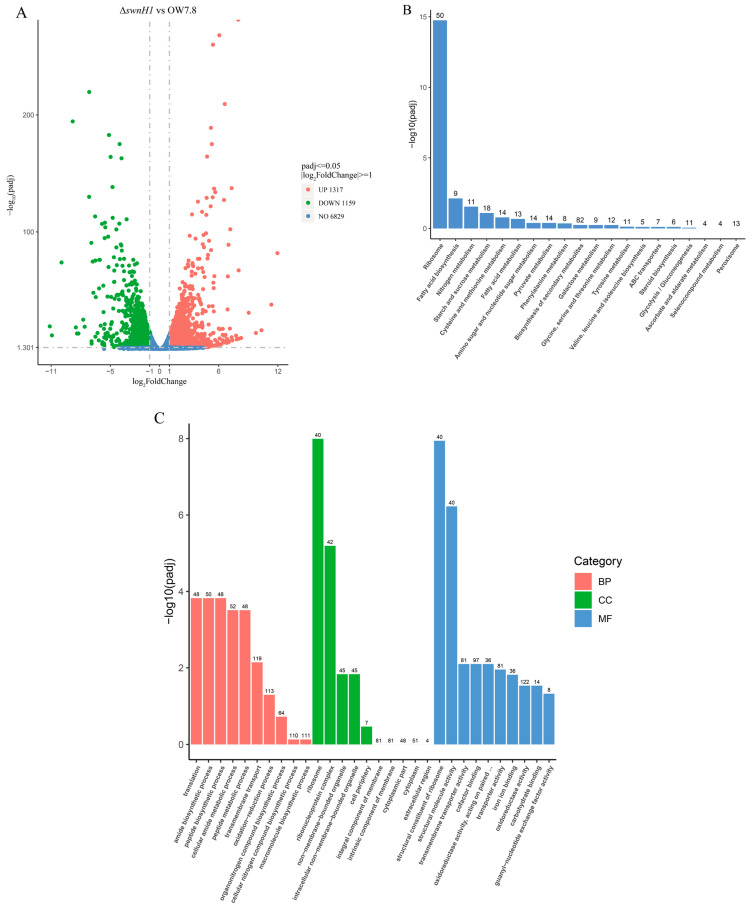
Transcriptome analysis between *A. oxytropis* OW 7.8 and Δ*swnH1*. (**A**) Volcano plot for differential comparison. (**B**) KEGG enrichment analysis and (**C**) GO functional classification annotation. BP: Biological Process; CC: Cellular Component; MF: Molecular Function.

There were no *swnH1* transcripts detected from Δ*swnH1* transcriptomics. Transcriptome sequencing of *A. oxytropis* OW 7.8 and Δ*swnH1* produced a total of 285,278,410 clean reads, amounting to 42.8 G. Gene differential expression was shown between *A. oxytropis* OW 7.8 and Δ*swnH1*. A total of 2310 DEGs were identified, of which 1087 (47.06%) were up-regulated and 1223 (52.94%) were down-regulated (Figure 9A). KEGG enrichment results indicated that the top two proportions of DEGs were related to ribosome and fatty acid biosynthesis, followed by nitrogen metabolism, starch and sucrose metabolism, cysteine and methionine metabolism, and fatty acid metabolism; a total of 315 metabolic pathways (Figure 9B) were obtained. In total, 48 DEGs involved in ribosomes were up-regulated and 2 were down-regulated; 1 DEG involved in fatty acid biosynthesis was up-regulate and 8 were down-regulated; 9 DEGs involved in nitrogen metabolism were up-regulated and 2 were down-regulated; 5 DEGs involved in starch and sucrose metabolism were up-regulated and 13 were down-regulated; 4 DEGs involved in cysteine and methionine metabolism were up-regulated and 10 were down-regulated; 4 DEGs involved in fatty acid metabolism were up-regulated and 9 were down-regulated.

The GO enrichment results indicated that the DEGs were involved in 250 biological processes, 52 cellular components, and 175 molecular functions. Processes with relatively great differences included translation, amide biosynthetic process, ribosomes, ribonucleoprotein complex, structural constituents of ribosomes, and structural molecule activity (Figure 9C). Specifically, 46 DEGs were up-regulated and 2 were down-regulated for translation; 48 DEGs were up-regulated and 2 were down-regulated for amide biosynthetic process; 39 DEGs were up-regulated and 1 was down-regulated for ribosomes; 41 DEGs were up-regulated and 1 was down-regulated for ribonucleoprotein complex; 39 DEGs were up-regulated and 1 was down-regulated for structural constituents of ribosomes; 39 DEGs were up-regulated and 1 was down-regulated for structural molecule activity.

Five DEGs closely related to SW synthesis were identified, among which the genes of *swnN*, *swnH2*, *swnK*, *swnR* and *LYS9* (*sac*) were down-regulated.

### 3.7. Metabolomic Analysis of A. oxytropis OW 7.8 and ΔswnH1

**Figure 10:**
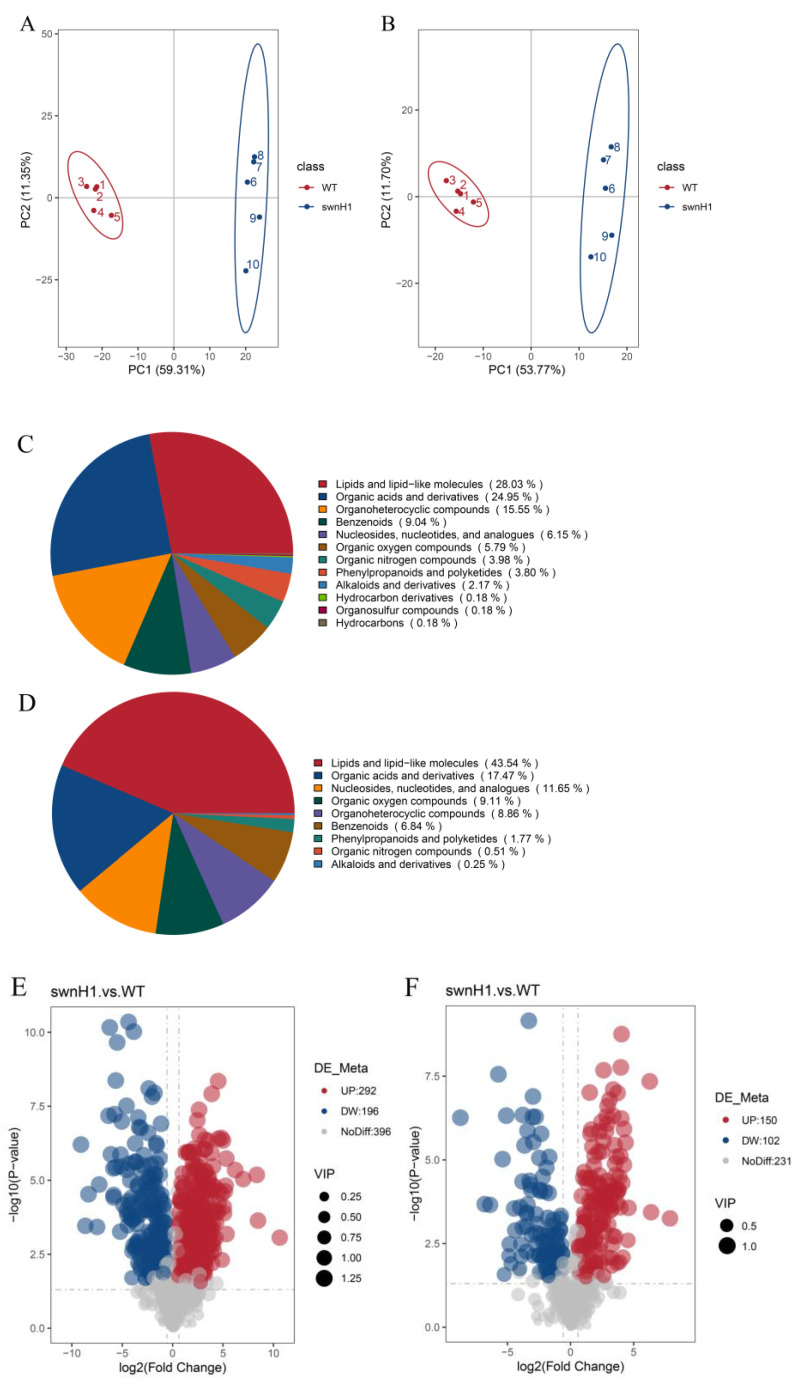

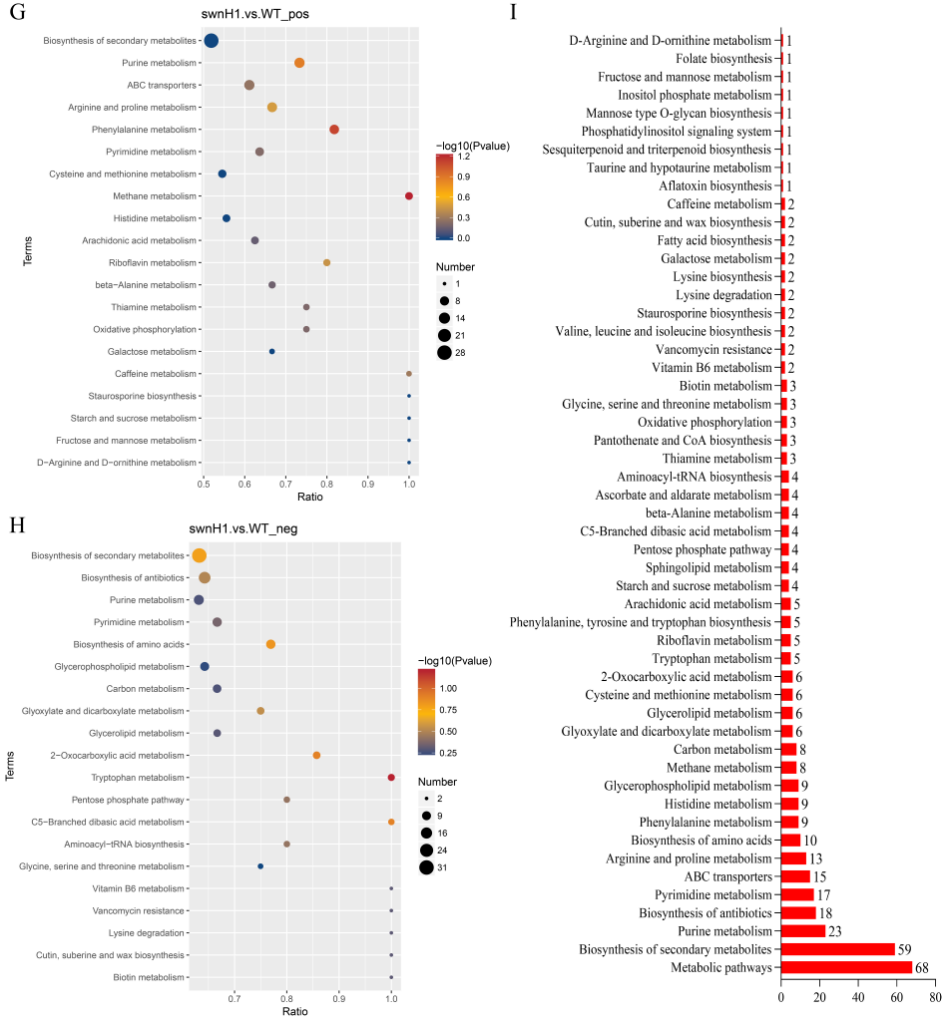
Metabolome analysis between *A. oxytropis* OW 7.8 and Δ*swnH1*. (**A**) Principal component analysis in positive ion mode. (**B**) Principal component analysis in negative ion mode. (**C**) Pie chart of metabolite classification in positive ion mode. (**D**) Pie chart of metabolite classification in negative ion mode. (**E**) differential metabolite volcano plot in positive ion mode. (**F**) differential metabolite volcano plot in negative ion mode. (**G**) Scatter plot of KEGG enrichment of differential metabolites in positive ion mode. (**H**) Scatter plot of KEGG enrichment of differential metabolites in negative ion mode. (**I**) KEGG enrichment analysis of DEMs.

The principal component analysis (PCA) of the metabolome data for *A. oxytropis* OW 7.8 and Δ*swnH1* is shown in Figure 10A,B, indicating differences in metabolites between Δ*swnH1* and *A. oxytropis* OW 7.8. In positive ion mode, the most abundant metabolites were lipids and lipid-like molecules (28.03%), followed by phenylpropanoids and polyketides (3.80%), and alkaloids and derivatives (2.17%). In negative ion mode, the most abundant metabolites were lipids and lipid-like molecules (43.54%), followed by organicheterocyclic compounds (8.86%), phenylpropanoids and polyketides (1.77%), and alkaloids and derivatives (0.25%) (Figure 10C,D). In positive ion mode, 488 DEMs were identified, with 292 up-regulated and 196 down-regulated. In negative ion mode, 252 DEMs were identified, with 150 up-regulated and 102 down-regulated (Table 2).

KEGG enrichment analysis was performed on 586 DEMs, with a total of 382 DEMs annotated and enriched into 52 metabolic groups, including 68 DEMs for metabolic pathways, 59 DEMs for biosynthesis of secondary metabolites, 23 DEMs for purine metabolism, 18 DEMs for biosynthesis of antibiotics, 17 DEMs for pyrimidine metabolism, and 15 DEMs for ABC transporters. (Figure 10I).

Six DEMs involved in SW synthesis were identified with P450, PKS-NRPS, saccharopine, lipopolysaccharide kinase, *L*-PA and α-aminoadipic, *L*-stachydrine being down-regulated, while *L*-proline was up-regulated.

### 3.8. Hypothesized SW Biosynthesis Pathway in A. oxytropis OW 7.8

**Figure 11:**
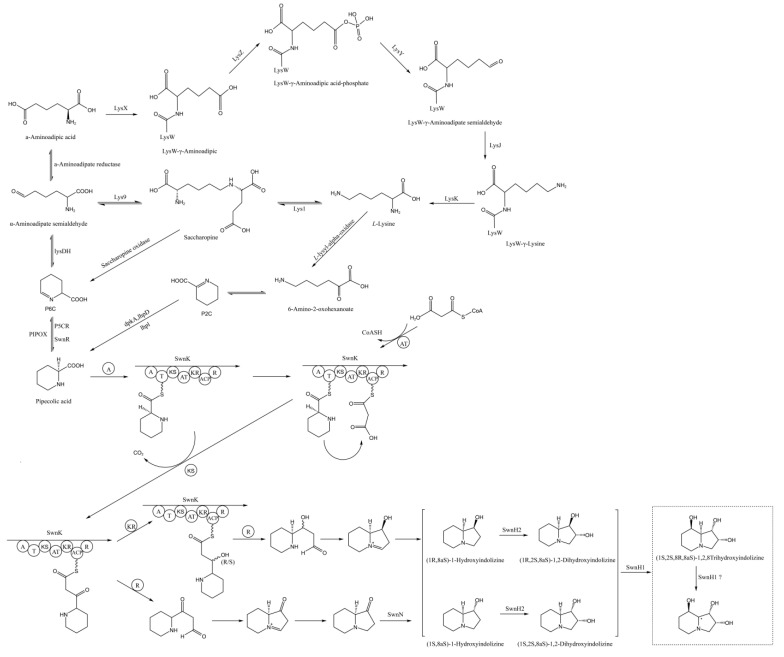
SW Biosynthesis Pathway in *A. oxytropis* OW 7.8. Note: LysX, LysZ, LysY, LysJ, and LysK are trypsin enzymes that can specifically cleave peptide bonds at different positions of lysine. SDH, LYS1, and LYS9 are saccharine reductases. lhpD/dpkA: Delta-1-piperideine-2-carboxylate reductase, lhpI: 1-piperideine-2-carboxylate reductase, PIPOX: *L*-PA oxidase.

The predicted SW biosynthesis pathway in *A. oxytropis* OW 7.8 is shown in Figure 11, starting from *L*-lysine artificially. The Sac enzyme catalyzes the synthesis of saccharopine from *L*-lys. Saccharopine was reduced to α-aminoadipic semialdehyde (α-aminoadipic semialdehyde was also formed from α-aminoadipic acid catalyzed by α-aminoadipate reductase). α-Aminoadipic semialdehyde cyclizes to form P6C (P6C was also formed from saccharopine catalyzed by saccharopine oxidase). P6C was then catalyzed by the P5CR enzyme or SwnR enzyme to form *L*-PA. There might be an alternative pathway synthesizing *L*-PA, in which *L*-lys was converted to 6-Amino-2-oxohexanoate catalyzed by *L*-lysyl-alpha-oxidase. 6-amino-2-oxohexanoate isomerizes to form P2C, which was subsequently catalyzed by the enzymes lhpD/dpkA (Delta-1-piperideine-2-carboxylate reductase) and lhpI (1-piperideine-2-carboxylate reductase) to produce *L*-PA.

*L*-PA and malonyl are converted to (8aS)-1-oxoindolizine, (1R,8aS)-1-hydroxyindolizine, or (1S,8aS)-1-hydroxyindolizine by the action of multifunctional SwnK protein. Subsequently, (8aS)-1-oxoindolizine is catalyzed by the SwnN enzyme to form (8aS)-1-hydroxyindolizine. (1R,2S,8aS)-1,2-dihydroxyindolizine or (1S,2S,8aS)-1,2-dihydroxyindolizine were synthesized from (1R,8aS)-1-hydroxyindolizine or (1S,8aS)-1-hydroxyindolizine catalyzed by SwnH2 enzyme. Finally, SW was synthesized from (1R,2S,8aS)-1,2-dihydroxyindolizine or (1S,2S,8aS)-1,2-dihydroxyindolizine catalyzed by SwnH1 enzyme.

## 4. Discussion

The nucleotide sequence identity of the *swnH1* gene between *A. oxytropis* OW 7.8 and *A. oxytropis* Raft River (KY365741.1) were 100%, both containing a 66 bp intron (located at 13152–13218 bp in *A. oxytropis* Raft River) and encoding 306 amino acids, indicating a close phylogenetic relationship [27]. In contrast, the homologous gene in *M. robertsii* has no intron and encodes 307 amino acids [35]. 

Endophytic fungus *A. oxytropis* OW 7.8 exhibits a slow growth rate and long growing period in vitro, with a very low homologous recombination rate, resulting in few Δ*swnH1* transformants after long-term exploration. During the knockout experiment, a total of five knockout mutant strains were screened, and they were all sequence verified correctly. No SW was detected from mycelia of Δ*swnH1* in our results, consistent with the result from that of *M. robertsii* with undetectable SW level in Δ*swnH1* [35], suggesting that the function of the *swnH1* gene promotes SW synthesis in these fungi. 

Five SW biosynthesis closely related genes, namely *swnN*, *swnR*, *swnK*, *swnH2*, and *sac,* were down-regulated in Δ*swnH1* compared with those in *A. oxytropis* OW 7.8. The up-regulation of *L*-Lys and down-regulation of saccharopine in Δ*swnH1* might be attributed to the reason for no production of saccharopine from α-aminoadipate semialdehyde via α-aminoadipic acid, which promotes the synthesis of *L*-lys from α-aminoadipic acid via α-aminoadipyl-6-phosphate and α-aminoadipate semialdehyde. Additionally, the up-regulation of the aspartate-semialdehyde dehydrogenase gene further enhanced the synthesis of *L*-lys from *L*-aspartate through multiple steps.

The previously predicted SW biosynthesis pathway in *A. oxytropis* OW 7.8 included the P6C and P2C branches [38]. In this study, the P6C branch in *A. oxytropis* OW 7.8 was refined, predicting that α-aminoadipic semialdehyde can also be produced from α-aminoadipic acid catalyzed by α-aminoadipate reductase, and P6C can also be formed from saccharopine catalyzed by saccharopine oxidase. The pathway from *L*-PA to SW was also refined, including the production of *L*-PA from (8aS)-1-indolizidinone, (1R,8aS)-1-hydroxyindolizine or (1S,8aS)-1-hydroxyindolizine by action of the SwnK protein, the production of (8aS)-1-oxoindolizine to (8aS)-1-hydroxyindolizine catalyzed by the SwnN enzyme, the production of (1R,2S,8aS)-1,2-dihydroxyindolizine or (1S,2S,8aS)-1,2-dihydroxyindolizine from (1R,8aS)-1-hydroxyindolizine or (1S,8aS)-1-hydroxyindolizine catalyzed by the SwnH2 enzyme, and the production of SW from (1R,2S,8aS)-1,2-dihydroxyindolizine or (1S,2S,8aS)-1,2-dihydroxyindolizine catalyzed by the SwnH1 enzyme. 

## 5. Conclusions

The *swnH1* gene of the endophytic fungus *Alternaria oxytropis* OW 7.8 isolated from *Oxytropis glabra* was cloned, and the gene knockout mutant Δ*swnH1* was first constructed. The colony morphology of Δ*swnH1* differed from that of *A. oxytropis* OW 7.8, appearing creamy yellow, with irregular shapes and a slower growth rate. Compared to *A. oxytropis* OW 7.8, no SW was detected in the mycelia of Δ*swnH1* cultured for 20 days, indicating that the function of the *swnH1* gene is to promote SW biosynthesis. Five DEGs and six DEMs closely associated with SW biosynthesis were identified by analyzing the data of the transcriptome and metabolome of *A. oxytropis* OW 7.8 and Δ*swnH1*. The SW biosynthesis pathway in *A. oxytropis* OW 7.8 was hypothesized and refined. The results provided a basis for in-depth study on the molecular mechanisms and metabolic pathway of SW in fungi, which would be beneficial for environmental protection and the sustainable use of grassland ecosystems.

## Figures and Tables

**Table 1:** Members of the SWN gene clusters and their predicted functions [27,35].

Gene	Encoding Product	Function Prediction
*swnA*	Aminotransferase	Catalyzing the synthesis of pyrroline-6-carboxylate (P6C) from *L*-lysine
*swnN*	Dehydrogenase or reductase	Catalyzing the synthesis of 1-hydroxyindolizine from 1-oxoindolizidine
*swnT*	Transmembrane transporter	Transport of SW
*swnH1*	Fe(II)/α-Ketoglutarate-dependent dioxygenase	Catalyzing the synthesis of SW from 1,2-dihydroxyindolizine
*swnH2*	Fe(II)/α-Ketoglutarate-dependent dioxygenase	Catalyzing the synthesis of 1,2-dihydroxyindolizine from 1-hydroxyindolizine
*swnK*	Multifunctional protein	Catalyzing the synthesis of 1-oxoindolizidine (or 1-hydroxyindolizine) from *L*-PA
*swnR*	Dehydrogenase or reductase	Catalyzing the synthesis of *L*-PA from P6C

**Table 2:** Screening of DEMs.

Screening Mode	Total of Metabolites	Total of DEMs	Up-Regulated	Down-Regulated
Positive	751	345	203	142
Negative	366	175	63	112

## Data Availability

The data presented in this study are available on reasonable request from the corresponding author.
